# Social inequalities in secondhand smoke exposure in children in Spain

**DOI:** 10.18332/tid/85717

**Published:** 2018-04-18

**Authors:** Maria J. López, Teresa Arechavala, Xavier Continente, Anna Schiaffino, Mónica Pérez-Ríos, Esteve Fernández

**Affiliations:** 1 Evaluation and Intervention Methods Service, Agència de Salut Pública de Barcelona, Barcelona, Spain; 2 CIBER de Epidemiología y Salud Pública (CIBERESP), Madrid, Spain; 3 Universitat Pompeu Fabra (UPF), Department of Experimental and Health Science, Barcelona, Spain; 4 Institut d’Investigació Biomèdica Sant Pau (IIB St. Pau), Barcelona, Spain; 5 Direcció General de Planificació en Salut, Departament de Salut Generalitat de Catalunya, Barcelona, Spain; 6 Epidemiology Unit, Galician Directorate for Public Health, Galician Health Authority, Xunta de Galicia, Santiago de Compostela, Spain; 7 Department of Preventive Medicine and Public Health, School of Medicine, University of Santiago de Compostela, Santiago de Compostela, Spain; 8 Cancer Prevention and Control Group, Institut d’Investigació Biomèdica de Bellvitge (IDIBELL), Barcelona, Spain; 9 Tobacco Control Unit, Cancer Control and Prevention Program, Institut Català d’Oncologia (ICO), Barcelona, Spain; 10 Department of Clinical Sciences, School of Medicine and Health Sciences, Universitat de Barcelona, Barcelona, Spain

**Keywords:** tobacco smoke pollution, secondhand smoke, children, inequalities

## Abstract

**INTRODUCTION:**

Children are particularly vulnerable to the health effects of secondhand smoke (SHS). The objectives of this study are to describe SHS exposure of children younger than 12 years in Spain and to identify potential social inequalities associated with SHS exposure.

**METHODS:**

A cross-sectional study was conducted in a representative sample of the population younger than 12 years in Spain. A computer-assisted telephone interview was conducted with parents or legal guardians in 2016, to assess the children’s SHS exposure at home, in the car, at school and at the nursery gates, in public transport, and during leisure time. The socio-demographic variables included were the child’s age and sex, the highest educational attainment at home, and occupational social class. Prevalence and 95% confidence intervals were calculated for SHS exposure in each setting and for overall exposure.

**RESULTS:**

In all, 71.8% of the children were exposed to SHS: 25.8% were exposed at home, 4.6% in the car, 8.2% in public transport, 31.9% at outdoor nursery or school gates, and 48% during leisure time. The higher the educational attainment at home, the lower the exposure (38.8% for primary school or lower, 28.7% for secondary school and 20.8% university level). The more deprived the social class, the higher the exposure (21.7% class I-II, 23.4% class III-IV and 31.1% class V-VII). SHS exposure in cars and overall exposure also decreased with higher educational achievement.

**CONCLUSIONS:**

In Spain, a large proportion of children are still exposed to SHS. Furthermore, there are clear social inequalities. To reduce SHS exposure, there is an urgent need for evidence-based interventions with an equity perspective.

## INTRODUCTION

Children are particularly vulnerable to the health effects of secondhand smoke (SHS), mainly due to their developing respiratory and immune systems and faster respiratory rate. In addition, infants can spend long periods in the arms or on the laps of their parents or caregivers, making them very close to the source of smoke^1^. According to some studies, children whose parents are smokers with low socioeconomic status (SES) might be at increased risk of SHS exposure in

the home^2^.

In 2006, a tobacco control law was implemented in Spain, banning smoking in workplaces and public places, except for hospitality venues^3^. This law was modified 5 years later, banning smoking in all hospitality venues. The 2011 law recognized that children were ‘a vulnerable group to be protected’ and established specific measures to protect them, such as the ban on smoking in outdoor children’s playgrounds^4^. However, the law did not cover some of the settings where children might spend most of their time, such as homes and cars.

Data on the prevalence of exposure among children in Spain are very scarce. So far, only the Spanish National Health survey (NHS) has reported some national data, but only in home and indoor public places. In 2011-2012, according to the Spanish NHS, 11.5% of children younger than 15 years were exposed to SHS at home and less than 1% in indoor public places^5^.

Information on SHS exposure of children at the national level, in a wider range of settings, so far has not been available. Therefore, the objectives of this study are to describe SHS exposure of children younger than 12 years in Spain and to identify potential social inequalities associated with SHS exposure.

## METHODS

This cross-sectional study was performed in a representative sample of the population younger than 12 years in Spain. The sample consisted of 2411 individuals and was proportional by geographical region, size of municipality of residence, sex, and age groups (0-1, 2-3, 4-5, 6-7, 8-9, 10-11 years).

A computer-assisted telephone interview was conducted with parents or legal guardians in 2016. Participants were contacted using randomly selected mobile phones (n=773) and landlines (n=1638). The selection of households was made proportionally according to previously established quotas.

Participants were asked about children’s SHS exposure at home, in the car, at school and nursery gates, on public transport, and during leisure time. To assess SHS exposure at home, we asked if any household member normally smoked inside the house and/or outdoors such as the terrace or balcony. SHS exposure in the car was determined from the time the child had spent in a private car while someone smoked in the last week (categorized as 0 min/day, 30 min/day, 31-60 min/day, >60 min/day, don’t know). The child was considered to be exposed in the car if someone had smoked ≥1 min/day in the child’s presence. SHS exposure on public transport was assessed by asking if the child had used a means of transport in which someone had smoked (yes/no) and if someone had done so at the transport stop (yes/no). Exposure at the school or nursery was determined by the following question: ‘During the last week, has anyone smoked at the entrance/exit door in their presence? (Yes/No; Does not go to school or nursery; Don’t know). In the case of leisure time outside the home, we asked about SHS exposure in different settings (bars, cafeterias, restaurants or terraces, leisure centers, parks, family or friends’ houses, and other places) during the last week (Yes/No; Don’t know). All the variables were dichotomized and overall exposure was defined as SHS exposure in at least one setting (Yes/No).

The socio-demographic variables considered were the child’s age and sex, and the highest educational attainment at home. We also included occupational social class, based on the National Classification of Occupations (NCO-2011) of the main earner at home^6^.

We calculated the prevalence and 95% confidence intervals (CI) for SHS exposure. We also performed analyses stratified by children’s age, sex, and also the educational attainment and social class of the main earner at home. The p-value for trend was also assessed. Finally, we fitted robust Poisson models for each setting and adjusted them by children’s sex, age, and studies of the main earner at home. All analyses were performed with Stata 13.1.

This study was approved by the ‘Parc de Salut Mar Clinical Research Ethics Committee’ and is registered under code 2015/6501/I.

## RESULTS

Half of the sample (51%) were boys. The highest educational attainment at home was university education in 50% of the families, and secondary school education in 40%. A total of 70.9% of the households had no smokers. Most respondents were 31-50 years old, and 61.8% of the survey respondents were women.

As shown in Tables 1A and 1B, 71.8% of the children were exposed to SHS. A total of 25.8% were exposed at home, 4.6% in the car, 8.2% on public transport (including stations), 31.9% in outdoor nursery or school gates and 48% during leisure time. The highest prevalence of exposure during leisure time was observed in outdoor terraces in bars, cafeterias and restaurants (26.0%, data not shown in Tables). An exposure gradient of SHS exposure at home was observed according to SES : the higher the educational level at home, the lower the exposure (38.8% in families with primary school or lower, 28.7% with secondary school and 20.8% with university level, p trend <0.001); conversely, the more deprived the social class, the higher the exposure (21.7% class I-II, 23.4% class III-IV and 31.1% class V-VII, p trend <0.001). SHS exposure in cars and overall SHS exposure also decreased as the educational attainment at home increased. The gradient observed according to education level remains in homes and cars, after adjusting by the other variables (Home: adjusted Prevalence Ratio, aPR=2.07, 95%CI: 1.24-3.44 primary studies, aPR=1.60, 95%CI: 1.04-2.44 secondary studies; Car: aPR=1.89, 95%CI: 1.58-2.26 primary studies, aPR=1.39, 95%CI: 1.19-1.63 secondary studies).

**Table 1A:** Prevalence and prevalence ratio of SHS exposure in different private settings by children’s sex and age, social class and education of the main earner at home, Spain, 2016

	*Home*						*Car*					
	*n*	%	*95% CI*	*p*^*b*^	*aPR*	*95% CI*	*n*	%	*95% CI*	*p*^*b*^	*aPR*	*95% CI*
**TOTAL**	622	25.8	24.1-27.6				109	4.6	3.8-5.5			
**Sex**												
Boys	319	25.9	23.6-28.5		1		58	4.8	3.7-6.1		1	
Girls	303	25.7	23.2-28.2		0.91	0.63-1.31	51	4.4	3.3-5.7		0.98	0.86-1.12
**Age (years)**										0.01		
0-3	188	25.5	22.5-28.8		1		27	3.7	2.5-5.3		1	
4-7	198	24.6	21.8-27.7		0.93	0.56-1.56	28	3.5	2.5-5.1		0.94	0.79-1.12
8-11	236	27.1	24.3-30.1		1.62	1.04-2.54	54	6.3	4.8-8.1		1.02	0.86-1.20
**Education of home main earner**				<0.001						0.002		
Primary	135	37.8	32.9-43.0		2.07	1.24-3.44	24	6.8	4.6-9.9		1.89	1.58-2.26
Secondary	274	27.8	25.1-30.7		1.60	1.04-2.44	51	5.3	4.1-6.8		1.39	1.19-1.63
University	212	20.0	17.7-22.5		1		34	3.2	2.3-4.5		1	
**Social class of the main earner^a^**				<0.001						0.09		
I-II	190	21.7	19.1-24.6				31	3.6	2.5-5.0			
III-IV	119	23.4	19.9-27.3				24	4.8	3.2-7.0			
V-VII	284	31.1	28.2-34.2				47	5.2	4.0-6.9			

aPR: adjusted Prevalence Ratio, CI: Confidence Interval

a Social class according to the National Classification of Occupations (NCO-2011) proposed by the Spanish Society of Epidemiology6 (I-II: Directors, managers and university professionals; III-IV: Intermediate activities and own account workers; V-VII: Manual workers)

b Mantel-Haenzel Test for linear trend

**Table 1B:** Prevalence and prevalence ratio of SHS exposure in different public settings by children’s sex and age, social class and education of the main earner at home, Spain, 2016

	*Public transport (including stations)*						*School & nursery gates*					
	*n*	%	*95% CI*	*p*^*b*^	*aPR*	*95% CI*	*n*	*%*	*95% CI*	*p*^*b*^	*aPR*	*95% CI*
**TOTAL**	192	8.2	7.2-9.4				760	31.9	30.0-33.8			
**Sex**												
Boys	85	7.1	5.8-8.7		1		391	32.3	29.7-35.0		1	
Girls	107	9.4	7.8-11.2		1.32	1.01-1.74	369	31.4	28.8-34.1		0.97	0.86-1.08
**Age (years)**				0.273						<0.001		
0-3	64	8.8	7.0-11.1		1		134	24.5	21.1-28.3		1	
4-7	67	8.6	6.8-10.8		0.97	0.70-1.35	295	37.3	34.0-40.7		1.49	1.25-1.77
8-11	61	7.3	5.8-9.3		0.82	0.58-1.14	331	38.7	35.5-42.0		1.55	1.31-1.84
**Education of home main earner**				0.267						<0.001		
Primary	34	10.0	7.3-13.7		1.28	0.88-1.88	108	33.2	28.3-38.5		1.07	0.89-1.29
Secondary	77	8.1	6.5-10.0		1.05	0.77-1.42	355	39.9	36.7-43.1		1.28	1.13-1.45
University	81	7.8	6.3-9.6		1		293	30.2	27.4-33.2		1	
**Social class of the main earner^a^**				0.07						0.04		
I-II	61	7.1	5.6-9.1				251	29.0	26.1-32.1			
III-IV	36	7.3	5.3-9.9				170	33.7	29.7-38.0			
V-VII	84	9.5	7.8-11.7				303	33.7	30.7-36.8			

	***Leisure time***^***a***^						***Overall exposure***^***b***^					
	***n***	**%**	***95% CI***	***p*^d^**	***aPR***	***95% CI***	***n***	***%***	***95% CI***	***p*^*d*^**	***aPR***	***95% CI***

**TOTAL**	1074	48.0	45.9-50.0				1637	71.8	69.9-73.6-			
**Sex**												
Boys	558	48.7	45.9-51.6		1		832	71.5	68.9-74.1		1	
Girls	516	47.1	44.2-50.1		0.97	0.89-1.06	805	72.0	69.3-74.6		1.01	0.96-1.06
**Age (years)**				0.09						0.011		
0-3	343	50.3	46.6-54.0		1		481	68.8	65.3-72.1		1	
4-7	363	48.1	44.5-51.6		0.95	0.86-1.06	542	71.3	68.0-74.4		1.04	0.97-1.11
8-11	368	45.8	42.4-49.3		0.91	0.82-1.01	614	74.7	71.6-77.6		1.08	1.01-1.15
**Education of home main earner**				0.371						<0.001		
Primary	162	48.1	42.8-53.4		1.04	0.92-1.19	259	76.2	71.4-80.4		1.12	1.04-1.20
Secondary	456	49.5	46.3-52.7		1.07	0.98-1.18	696	74.4	71.5-77.1		1.09	1.03-1.15
University	452	46.5	43.4-49.6		1		677	68.0	65.0-70.8		1	
**Social class of the main earner^c^**				0.95						0.01		
I-II	388	48.3	44.8-51.7				574	69.7	66.5-72.8			
III-IV	226	46.8	42.4-51.3				338	69.7	65.5-73.6			
V-VII	410	48.4	45.1-51.8				651	75.3	72.3-78.0			

aPR: adjusted Prevalence Ratio, CI: Confidence Interval

a Includes bars, cafes, restaurants or terraces, leisure centers, parks, family or friends’ houses, or other places

b Exposure in at least one of the private and public settings studied

c Social class according to the National Classification of Occupations (NCO-2011) proposed by the Spanish Society of Epidemiology6 (I-II: Directors, managers and university professionals; III-IV: Intermediate activities and own account workers; V-VII: Manual workers)

d Mantel-Haenzel Test for linear trend

## DISCUSSION

In Spain, nearly 3 out of 4 children were exposed to SHS. Approximately 50% of the children were exposed during leisure time, more than 30% at the school gates and 26% were exposed at home. Overall SHS exposure was higher in children whose parents had primary and secondary school education compared to children whose parents had university education. These differences were especially clear in the case of SHS exposure at home and in cars, for primary school education (aPR=2.07 and 1.89, respectively).

When the different settings were taken into account, SHS exposure affected more than 70% of the children. Therefore, as there is no level of SHS exposure that can be considered safe, it is important to include all these settings in public health studies. In a study carried out on adults in Spain in 2011^7^, we found a prevalence of overall SHS exposure (including home, work, transport, and leisure time) of 45.2%, indicating that children, who are especially vulnerable, were even more exposed than adults.

One of the settings that has been most widely studied is the home. In this setting, the prevalence of exposure in our study was 26%. Furthermore, according to a recent study^8^, the level of exposure in this setting might be very high, with nicotine concentration levels similar to those found in public places before the implementation of the tobacco control laws. Therefore, SHS exposure at home might have an important public health impact.

The inequalities observed with educational attainment were also found for social class. This finding is consistent with previous studies. A study carried out in Denmark showed that children were 11 times more likely to be exposed to SHS at home if the parents had a very low education level than if they were highly educated^9^. Another study carried out in Australia showed that the proportion of children who lived with a smoker declined between 2001 and 2010 in all social groups except the most disadvantaged households^10^. The results of another study carried out in Germany^11^ showed that 0-6 years old children with a low SES were more frequently exposed to SHS in the parental home (19.4% for low SES, 4.7% for medium SES, and 1.7% for high SES).

In our study, SHS exposure in cars was 4.6%, and a trend was also observed according to educational attainment at home. This prevalence is slightly higher than that registered in an observational study in Barcelona^12^ in which 2.2% of passengers younger than 14 years were exposed to SHS in vehicles. The inequalities found are also consistent with a recent study showing that SHS exposure of children traveling in cars that were registered in the most disadvantaged areas of Montreal were more likely to be exposed than children traveling in cars registered in the most advantaged areas^13^.

One of the potential limitations of this study is the use of a non-validated questionnaire. However, the design of the questionnaire was based on a previous questionnaire^7,14,15^, used to assess SHS in adults and was adapted ad-hoc to our population. In addition, a pilot test was conducted on 30 participants to minimize its potential limitations. The use of a questionnaire could also represent information bias since the respondents may not know if the children had been exposed when they were not with them. A desirability bias, with some parents underreporting the real SHS exposure, might also be possible. Finally, we do not have information about potential SHS exposure from external smokers in multi-unit housing, which may underestimate SHS exposure at home.

A strength of the study is that it was conducted in a representative national sample of children younger than 12 years in Spain. This is also the first study to show data on SHS exposure of children in multiple settings at the national level, as most previous studies have focused on homes or other selected settings. Finally, our study included two different, and very widely used, socioeconomic indicators to assess inequalities–educational level and social class–both of which showed the same pattern.

## CONCLUSIONS

This study shows that, despite the laws, a large proportion of children might still be exposed to SHS. Furthermore, there are clear social inequalities. Therefore, there is a need for evidence-based interventions with an equity perspective to reduce SHS exposure of children.

## CONFLICTS OF INTEREST

Authors have completed and submitted the ICMJE Form for Disclosure of Potential Conflicts of Interest and none was reported.
